# A commentary on ‘Effect of transarterial chemoembolization as postoperative adjuvant therapy for intermediate stage hepatocellular carcinoma with microvascular invasion: a multicenter cohort study’

**DOI:** 10.1097/JS9.0000000000000891

**Published:** 2023-11-16

**Authors:** Xin Zhao, Tianyang Mao, Qingyun Xie, Fengwei Gao, Hong Wu

**Affiliations:** a Department of Hepato-Pancreato-Biliary Surgery, The People’s Hospital of Leshan, Leshan; b Liver Transplantation Center, State Key Laboratory of Biotherapy and Cancer Center, West China Hospital, Sichuan University and Collaborative Innovation Center of Biotherapy, Chengdu, People’s Republic of China

*Dear Editor*,

We recently reviewed the multicenter retrospective study by Xiang *et al*.^1^ on the efficacy of adjuvant transarterial chemoembolization (TACE) following the radical resection of intermediate stage hepatocellular carcinoma with microvascular invasion (MVI). The study incorporated 246 patients: 109 in the surgical resection combined with the TACE group and 137 in the surgical group alone. Results demonstrated the combined surgical resection and TACE group had improved recurrence-free survival (RFS) and overall survival (OS) than the surgical group. The findings suggest that adjuvant TACE therapy is beneficial for postoperative treatment of intermediate stage hepatocellular carcinoma in Barcelona with MVI. While the study offers clinical insights for postoperative treatment of intermediate stage hepatocellular carcinoma with MVI, further discussions on several points are warranted.

Firstly, regarding patient inclusion criteria, the authors seem to overlook the impact of postoperative adjuvant use of tyrosine kinase inhibitors (TKI) or immune checkpoint inhibitor (ICI) on RFS and OS. Studies by Li *et al*.^2^ indicate that immune ICI usage, either alone or with TKI, can enhance RFS in patients with recurrent hepatocellular carcinoma post-radical resection. Hence, such patients should not be part of the study. Moreover, while the data mentions the proportion of hepatitis patients in both groups without showing differences, it does not compare antiviral therapy results between them. Numerous past studies validate that postoperative antiviral therapy for HBV-related hepatocellular carcinoma can prevent postoperative recurrence^3,4^. The influence of varying postoperative antiviral therapies on RFS and OS in both groups remains unclear in this study.

Secondly, concerning hepatectomy surgical methods, the baseline data mentions no disparity between anatomical and nonanatomical resections but does not detail surgical procedures, such as the prevalence of laparotomy, laparoscopy, and robot surgery in both groups. While existing research suggests similar long-term outcomes between minimally invasive hepatectomy and open hepatectomy, high-quality randomized control trial evidence is lacking^5,6^. Some research has assessed the efficacy of postoperative adjuvant TACE for intermediate stage hepatocellular carcinoma with MVI^7^, but the relation between MVI severity and TACE efficacy remains undefined. Elaborating on the therapeutic impact of TACE on M1 and M2 microvascular invasions would enhance the study’s rigor.

Lastly, in terms of outcome indicators, the authors did not specify postoperative recurrence treatment strategies for both groups. This omission might influence the OS results, potentially obscuring the genuine efficacy of postoperative adjuvant TACE. Prioritizing RFS and time to recurrence (TTR) as primary observation outcomes, with OS as secondary, might yield more accurate conclusions.

In summary, we appreciate the valuable clinical insights provided by the authors on postoperative treatment for intermediate stage hepatocellular carcinoma. Their conclusions are pivotal for postoperative adjuvant TACE in treating intermediate stage hepatocellular carcinoma with MVI. The study also offers direction for future research in postoperative adjuvant therapies for intermediate stage hepatocellular carcinoma. Moving forward, our focus should be on extending the RFS and OS for liver cancer patients with high recurrence risk, ultimately benefiting them.

## Ethical approval

Not applicable to this study.

## Sources of funding

This research did not receive any specific grant from funding agencies in the public, commercial, or not-for-profit sectors.

## Author contribution

X.Z. and T.M: study design and writing; Q.X. and F.G.: critical review; H.W.: study supervision.

## Conflicts of interest disclosure

No conflicting relationship exists for any of the authors.

## Research registration unique identifying number (UIN)

1. Name of the registry: not applicable.
2. Unique identifying number or registration ID: not applicable.
3. Hyperlink to your specific registration (must be publicly accessible and will be checked): not applicable.

## Guarantor

Prof. Hong Wu, e-mail: wuhong@scu.edu.cn, Liver Transplantation Center, No. 37, Guoxue Lane, Wuhou District, Chengdu, Sichuan Province, People’s Republic of China.

## Date availability statement

All data generated or analyzed during this study are included in this article. The data are available from the corresponding author upon reasonable request.
